# Association of elevated reactive oxygen species and hyperthermia induced radiosensitivity in cancer stem-like cells

**DOI:** 10.18632/oncotarget.21678

**Published:** 2017-10-09

**Authors:** Qibin Fu, Tuchen Huang, Xudong Wang, Chunyang Lu, Feng Liu, Gen Yang, Yugang Wang, Biao Wang

**Affiliations:** ^1^ Sino-French Institute of Nuclear Engineering and Technology, Sun Yat-sen University, Zhuhai 519082, P. R. China; ^2^ State Key Laboratory of Nuclear Physics and Technology, School of Physics, Peking University, Beijing 100871, P. R. China

**Keywords:** cancer stem-like cells, hyperthermia, radiosensitivity, reactive oxygen species

## Abstract

Cancer stem-like cells (CSCs) are the principal causes of tumor radio-resistance, dormancy and recurrence after radiotherapy. Clinical trials show hyperthermia (HT) might be a potent radiation sensitizer. In this study, CSCs were found to be more susceptible to radiation when combined with HT treatment. Treated cells showed significantly reduced self-renewal, cell survival and proliferation *in vitro*, as well as significant reduced tumor formation *in vivo*. Further study demonstrated that the radiosensitization effect was associated with increased intracellular reactive oxygen species (ROS) level in CSCs, confirmed by modifying redox status in CSCs bidirectionally. Pharmacologic depletion of glutathione by buthionine sulphoximine mimicked HT induced radiosensitivity in CSCs. Antioxidant N-acetylcysteine could efficiently rescue HT induced radiosensitivity in CSCs. To our knowledge, this may be the first report suggesting the association between elevated intracellular ROS level and HT induced radiosensitization in human breast CSCs and pancreatic CSCs, which might provide new strategy for improving CSCs radiosensitivity.

## INTRODUCTION

Accumulating data have indicated the existence of cancer stem-like cells (CSCs) in multiple solid tumors [1–3]. CSCs are a subset of tumor cells that have the ability to self-renewal and generate diverse tumor cells [2]. These cells are currently believed to be responsible for treatment failures because of their resistance to conventional treatments including ionizing radiation (IR) [4–6] and chemotherapy [7–9]. The mechanisms are suggested to be high free radical scavenger level [5, 10], low proteasome activity [11], preferentially activated DNA damage checkpoints [6], and expression of the ATP-binding cassette B5 (ABCB5) multi-drug resistance proteins [7].

Extensive clinical studies show hyperthermia (HT) can significantly improve both local tumor control and survival after radiation therapy, without major in side-effects [12–14]. HT therapy, through increasing the temperature of tumor-loaded tissue to 40–43°C, is applied as an adjuvant to radiotherapy and chemotherapy [15–17]. The interaction between IR and HT depends on many factors, including the temperature, the duration time, sequence and time interval between the two modalities [16]. Generally, higher temperature and longer exposure time will yield better the effect [16, 18]. Although the effects produced by the sequence between IR and HT may vary with different tumor types, combination of IR and HT at the same time kills the most tumor cells [18–19]. Moreover, as the time interval increases, the radiosensitization by heat could hardly be observed.

Previous studies show that HT acts as a cancer treatment for direct cell killing, radiosensitization, and promotion of tumor reoxygenation [16, 20]. Utilizing hyperthermia as a radiosensitizer, would reduce the dose of radiation, which in turn decrease toxicity to normal tissues. Recently, thermal radiosensitization of CSCs is suggested to result to inhibition of the repair of radiation-induced DNA double-strand breaks (DSBs) [21] or suppression of radiation-induced AKT activation and proliferation [22]. However, the mechanisms of HT induced radiosensitization of CSCs are still unclear.

In this study, HT induced radiosensitization in breast CSCs and pancreatic CSCs were observed *in vivo* and *in vitro*. We also found that the radiosensitization was associated with increased intracellular ROS level in CSCs. The association was further confirmed by modifying redox status in CSCs bidirectionally. To our knowledge, this may be the first report shedding light on elevated intracellular ROS level playing critical roles in HT induced radiosensitization in human breast CSCs and pancreatic CSCs, which provides new strategy for improving CSCs radiosensitivity.

## RESULTS

### Isolation and characterization of breast CSCs and pancreatic CSCs

**Figure 1:**
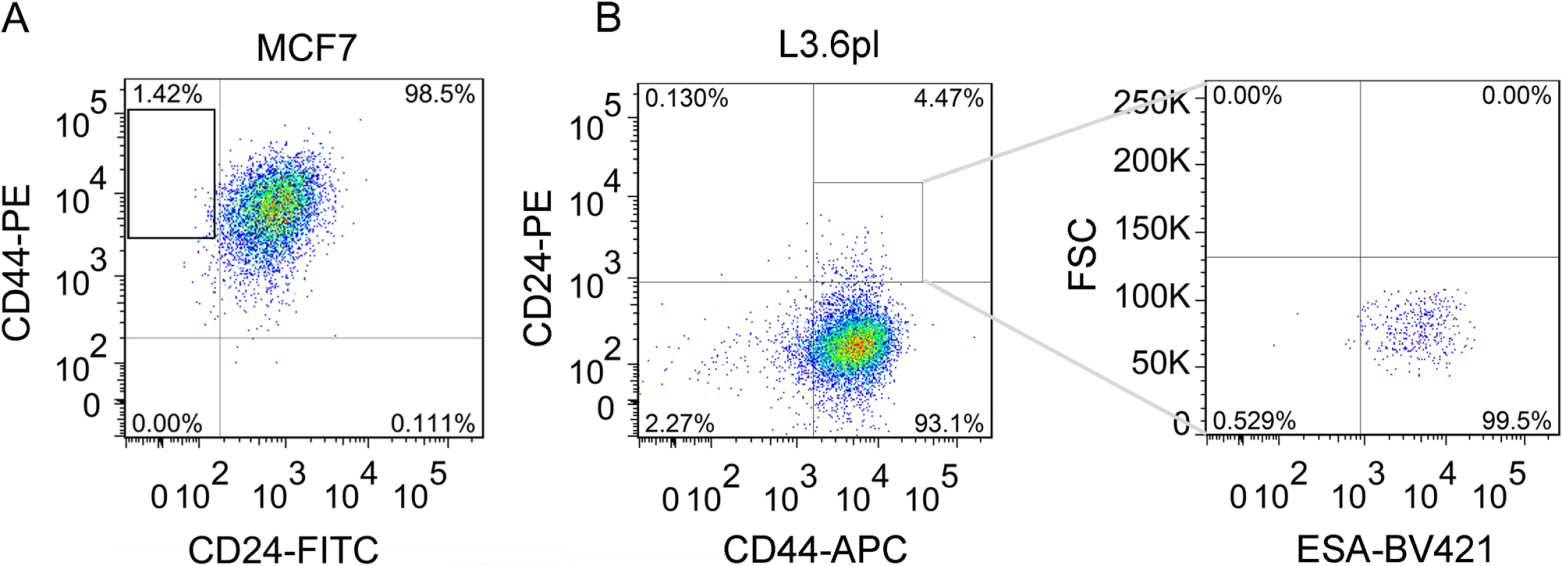
Isolation of breast CD44^+^CD24^-^ CSCs and pancreatic CD44^+^CD24^+^ESA^+^ CSCs from MCF7 and L3.6pl cells by flow cytometry (**A**) Typical proportion of CD44^+^CD24^–^ cells in MCF7 cells. The isolated CD44^+^CD24^–^ CSCs were obtained as shown in the frame of A and cultured 1d for re-analysis by flow cytometry (Supplementary Figure 1A). (**B**) Fluorescence activated cell-sorting (FACS) analysis to measure CD44 and CD24 expression of L3.6pl cells (Left) and the patterns of ESA staining of CD24^+^CD44^+^ cells as shown in the frame of panel B Left (Right).

We isolated CD44^+^CD24^–^ cells by flow cytometry as described previously [23] and the percentage of CD44^+^CD24^–^ cells were about 1.4% (Figure 1A). In addition, the isolated CD44^+^CD24^–^ cells were reanalyzed for the purity, which was 99% (Supplementary Figure 1A). As shown in Supplementary Figure 1B, MCF7 cells, which were mainly CD24^+^, exhibited a more differentiated morphology, consistent with their luminal-type classification [24–25], while CD44^+^CD24^–^ cells were spindle-like.

**Figure 2:**
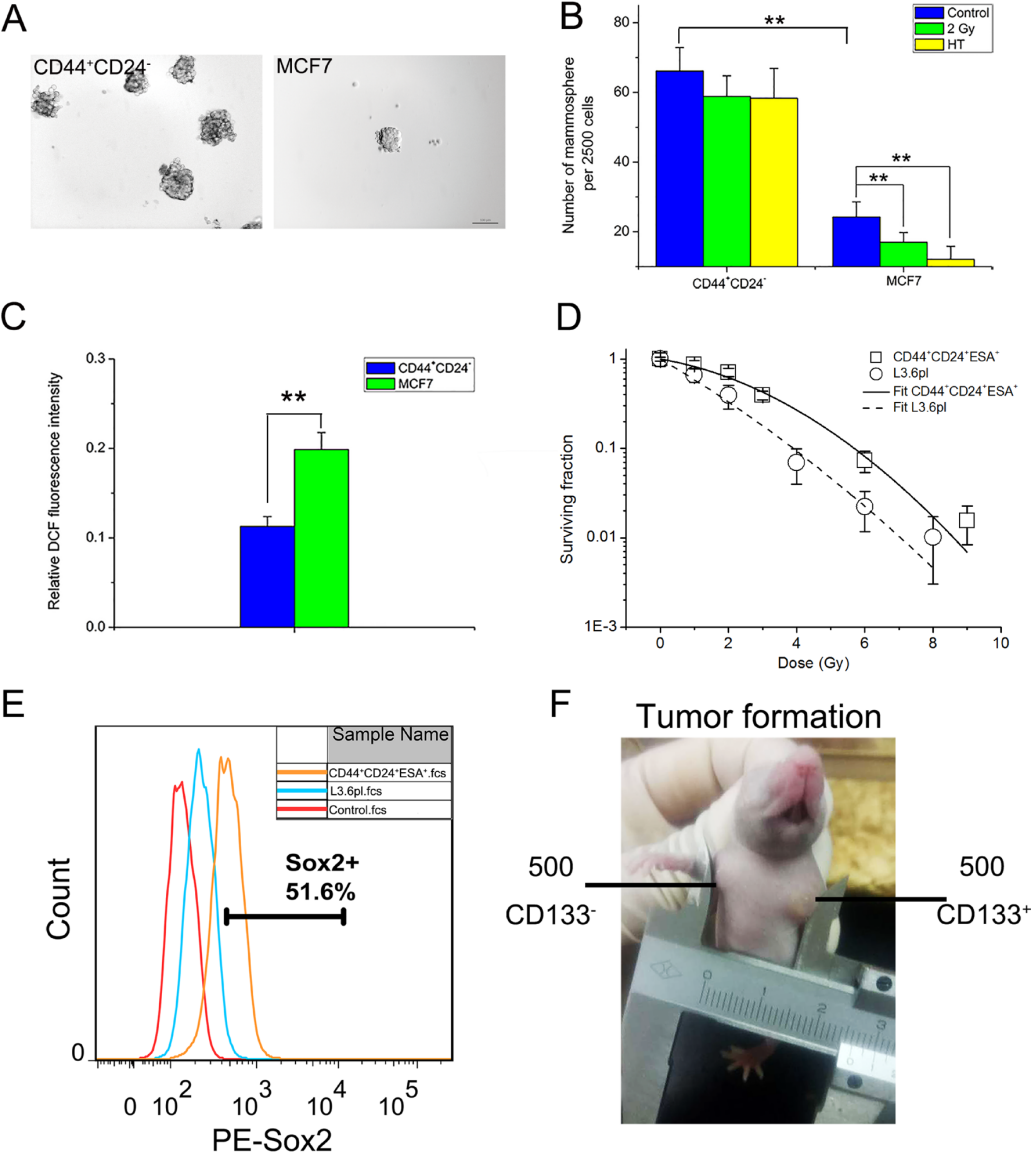
Identification of sorted breast CSCs and pancreatic CSCs *in vitro* and *in vivo* (**A**) Typical mammosphere formation of CD44^+^CD24^–^ cells and MCF7 cells imaged by 10x objective (Bar = 100 μm). (**B**) Quantification of mammospheres derived from CD44^+^CD24^–^ cells and MCF7 cells at day 7 after sham-treated, HT (43°C for 2 hours) or 2 Gy treatment. (**C**) Intracellular ROS concentrations of CD44^+^CD24^–^ cells and MCF7 cells. (**D**) Clonogenic survival assay in cells derived from CD44^+^CD24^+^ESA^+^ cells (squares) and L3.6pl cells (circles). To determine surviving fractions, counts were normalized using the plating efficiency of the unirradiated corresponding control. The survival curves of CD44^+^CD24^+^ESA^+^ cells (solid line) and L3.6pl cells (dashed line) were fitted using LQ model respectively. (**E**) FACS analysis to measure Sox2 expression of L3.6pl cells and CD44^+^CD24^+^ESA^+^ cells. (**F**) Representative experiment depicting tumor formation in a mouse at the injection site of 500 CD133^+^ cells, with no tumor formation seen at the injection site of 500 CD133^-^ cells. The results are presented as the mean ± SD, as determined from three independent experiments. ^**^P < 0.01.

Presently, CD44^+^CD24^–^ cells showed ~3-fold increase in mammosphere formation compared with MCF7 cells (Figure 2A and 2B), indicating stronger capacity in self-renewal. Moreover, CD44^+^CD24^–^ cells also showed much stronger mammosphere-forming capability after HT or IR treatment than MCF7 cells (Figure 2B). The results demonstrated that CD44^+^CD24^–^ cells are much more resistant to stresses including radiation and HT than MCF7 cells. Furthermore, consistent with the previous report [10], around half of ROS level in CD44^+^CD24^–^ cells were detected compared to MCF7 cells (Figure 2C). Taken together, the identification of CD44^+^CD24^–^ CSCs was in accordance with previous reports.

Likewise, the pancreatic CSCs were isolated through flow cytometry. As CD44^+^CD24^+^ESA^+^ or CD133^+^ pancreatic cancer cells exhibited stem cell properties [26–27], flow cytometry demonstrated the presence of a rare CSCs population. The proportion of such triple positive (CD44^+^CD24^+^ESA^+^, Figure 1B) or CD133^+^ (data not shown) subpopulations was about 4%. Actually, the small subpopulations (about 0.7%) in which the two kinds of CSCs markers overlap were sorted for the experiment (Supplementary Figure 2B).

The responses of CD44^+^CD24^+^ESA^+^ cells and L3.6pl cells to radiation were compared through clonogenic assay. To give quantitative description of the dose-response curve, the linear-quadratic (LQ) model was used [28]. For CD44^+^CD24^+^ESA^+^ cells, α is 0.148, β is 0.045, mean surviving fraction at 2 Gy [SF2Gy] = 0.62. For L3.6pl cells, α is 0.514, β is 0.02, mean surviving fraction at 2 Gy [SF2Gy] = 0.33 (Figure 2D). The results showed that CD44^+^CD24^+^ESA^+^ cells were more radioresistant than L3.6pl cells. Furthermore, in comparison to L3.6pl cells, CD44^+^CD24^+^ESA^+^ cells showed high expression of Sox2 (Figure 2E), which is a key factor involved in CSCs maintenance [29]. To investigate whether sorted CSCs display long-term tumorigenic potential, we evaluated their ability to generate tumors after serial transplantations. As shown in Table 1, CD133^+^ cells showed enhanced tumorigenic potential than CD133^–^ cells (*P* < 0.05). As few as 500 CD133^+^ cells are capable of generating visible tumors after 40 days. In contrast, no visible tumors were observed with CD133^-^ cells under the same conditions (Figure 2F). Taken together, sorted CD133^+^ cells or CD44^+^CD24^+^ESA^+^ cells showed the enhanced tumorigenic potential, increased expression of the stemness related molecule Sox2 and highly resistant to radiation, displaying their stem cell properties as previous works reported.

**Table 1:** Tumor formation ability of sorted pancreatic CSCs

Cell no.	500	1000	5000
CD133^+^	6/6	5/5	5/5
CD133^-^	0/6	1/5	3/5

Assayed for the ability to form tumors after injection into the mice at 500, 1000, and 5000 cells per injection. Data are expressed as number of tumors formed/number of injections.

### The hyperthermia sensitized breast CSCs and pancreatic CSCs to radiation

Due to the resistance to radiation therapy, CSCs contribute to tumor progression [6]. We investigated the hyperthermia ability in improving the radiosensitivity of breast CSCs and pancreatic CSCs by assessing mammosphere formation and colony formation, two indicators of the self-renewal capacity of CSCs *in vitro*.

**Figure 3:**
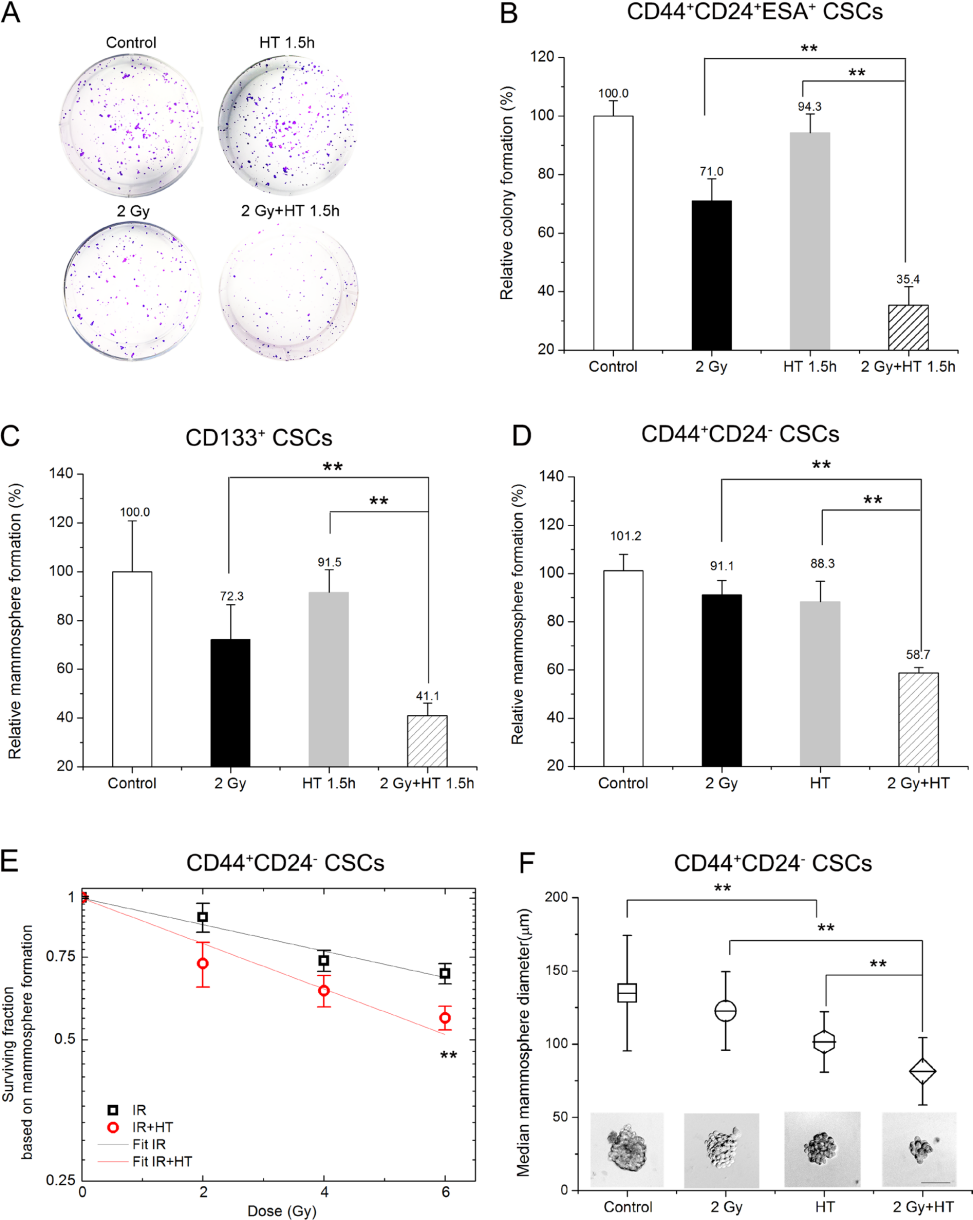
Hyperthermia sensitized breast CSCs and pancreatic CSCs to radiation (**A–B**) Representative images of colonies derived from CD44^+^CD24^+^ESA^+^ CSCs after sham-treated, HT (43°C for 1.5 hours), 2 Gy or 2 Gy+HT treatment (A) and quantification of colonies (B). (**C**) Quantification of mammospheres derived from CD133^+^ CSCs at day 7 after sham-treated, HT (43°C for 1.5 hours), 2 Gy or 2 Gy+HT treatment. (**D**) Statistics in mammosphere formations derived from CD44^+^CD24^-^ CSCs at day 7 after sham-treated, HT (43°C for 2 hours), 2 Gy or 2 Gy+HT treatment. (**E**) The mammosphere survival of CSCs after IR alone (squares) or IR+HT (circles) treatments. The dose-response curve of IR alone (black line) or IR+HT (red line) was fitted using the linear part of LQ model. (**F**) Representative images of mammosphere derived from CD44^+^CD24^-^ CSCs after different treatments. Bar = 100 μm (bottom) and mean values for mammosphere diameters of the indicated treatment groups. At least 40 mammospheres were measured in each group. The results are presented as the mean ± SD, as determined from three independent experiments. ^*^P < 0.05, ^**^P < 0.01.

In order to investigate radiosensitization by HT, the conditions of treatments had been optimized. A clinically relevant dose of 2 Gy was chosen for initial studies. 43°C was chosen as the heating temperature. The optimal duration of heating was 1.5 hours for pancreatic CSCs and 2 hours for breast CSCs. HT treatment procedure was performed immediately after irradiation. As shown in Figure 3A–3C, HT alone had little effect on pancreatic CSCs colony formation and mammosphere formation. IR alone attenuated colony formation and mammosphere growth by about 29%, but still a significant number of CSCs remained and were able to form colonies or mammospheres (Figure 3B and 3C). The most effective treatment was HT combined with IR, which could reduce pancreatic CSCs colony formation and mammosphere formation by additional 35% and 31% compared to IR alone (Figure 3B and 3C), respectively. For an intuitive description of the relative colony formation, the value of IR alone multiplied by that of HT alone was 71.0% × 94.3%, namely 67.0%, while the value for IR+HT was 35.4%. Obviously, the combined effect was greater than the additive effect, with less colony formation capability (Figure 3B). Furthermore, we determined whether the treated CSCs retained their tumorigenic properties after IR+HT treatment *in vivo*. As shown in Table 2, a significant decrease in tumor formation was observed after IR+HT treatment compared to IR alone. The results demonstrate HT radiosensitizes CSCs *in vitro* and *in vivo*.

**Table 2:** Tumor formation ability of sorted pancreatic CSCs after indicated treatments

Control	IR	HT	IR+HT
6/6	6/6	5/6	2/6

5000 cells after indicated treatments were injected into the mice. The tumor formation were evaluated at 40 d post cell implantation. Data are expressed as number of tumors formed/number of injections.

Additionally, similar results were also illustrated in breast CSCs. As is shown in Figure 3D, treatment with HT alone or IR alone had limited impact on mammosphere formation of CSCs compared to the sham-treated cells. However, IR+HT treatment significantly reduced mammosphere formation compared to IR alone (58.7% ± 2.3% in IR+HT group *versus* 91.1% ± 5.9% in IR group), indicating loss of self-renewal potential. Actually, treatment with IR+HT significantly decreased mammosphere-forming ability in CSCs compared to IR alone across the entire measured radiation dose range (2–6 Gy) (Figure 3E). Even at 12 Gy, the surviving fractions based on mammosphere formation were 42.0% ± 0.6% for IR group *versus* 35.6% ± 1.9% for IR+HT group. In order to analyze quantitatively, the dose-response curves were fitted by LQ model. For IR, α is 0.08341, β is –0.0034, for IR+HT, α is 0.16175, β is –0.01079. The ratio of these two slopes is 1.94 (the ratio of these two slopes is 1.83 when fitted by the linear response), showing a considerable enhancement in cell killing with hyperthermia. In addition, IR+HT significantly decreased average mammosphere size compared with IR alone (the diameter is 81.4 ± 22.9 μm for IR+HT *versus* 122.6 ± 26.7 μm for IR alone) (Figure 3F). Together, these studies demonstrate that IR combined HT is more effective than radiation alone in reducing the self-renewal capacity of CSCs.

### The addition of hyperthermia to radiation significantly reduced CSCs proliferation and viability

**Figure 4:**
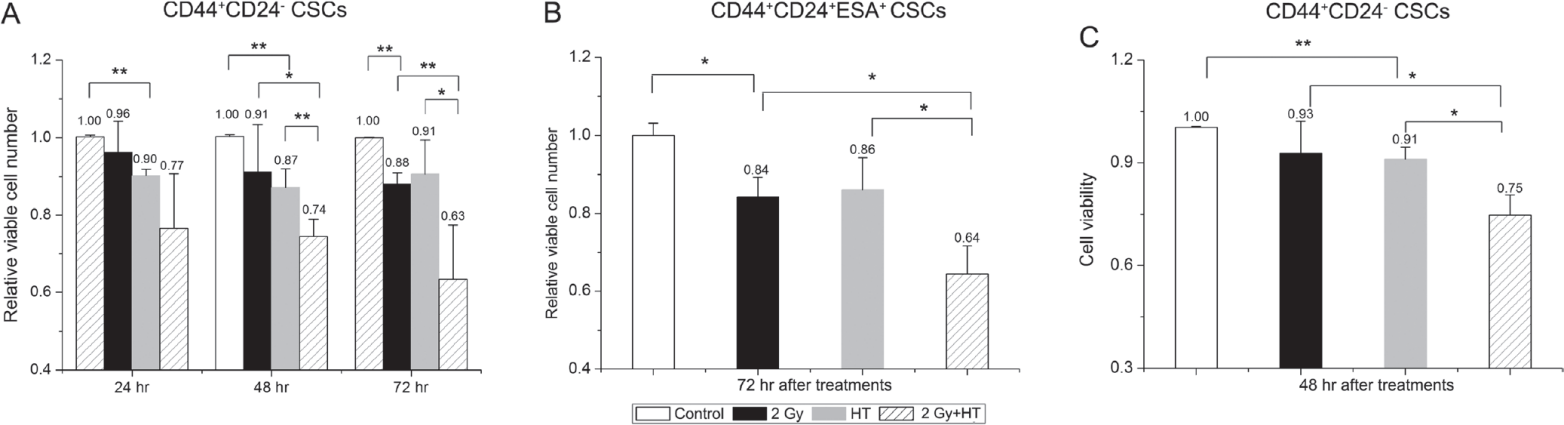
The addition of hyperthermia to radiation significantly reduced cell proliferation and viability in breast CSCs and pancreatic CSCs (**A**) The surviving fraction of CD44^+^CD24^–^ CSCs based on cell number counting at 24, 48, 72 hours after indicated treatments. (**B**) The surviving fraction of CD44^+^CD24^+^ESA^+^ CSCs based on cell number counting at 72 hours after indicated treatments. (**C**) The surviving fraction of CD44^+^CD24^–^ CSCs based on cell viability was detected by CCK-8 at 48 hours post indicated treatments. The results are presented as the mean ± SD, as determined from three independent experiments. ^*^P < 0.05, ^**^P < 0.01.

We next assessed the effect of hyperthermia on survival and proliferation in breast CSCs and pancreatic CSCs. As shown in Figure 4A, IR alone had no significant impact on cell number compared to control cells until 72 hours, on the contrary, HT alone significantly reduced survival fraction based on cell number compared with sham-treated cells (Figure 4A). However, treatment with combined IR and HT significantly reduced breast CSCs survival by additional 13% at 48 hours and 28% at 72 hours compared to HT alone respectively (Figure 4A). Additionally, a significant decrease in cell number at 72 hours was also observed in heated-irradiated pancreatic CSCs when compared to heated CSCs or irradiated CSCs (the relative viable cell number of HT alone and IR alone were 86.1% ± 8.3% and 84.2% ± 5.1% *versus* 64.4% ± 7.2% for IR+HT, *P* < 0.05, Figure 4B).

To determine whether the reduction in cell number reflected changes in the cell viability, we assessed cell viability by CCK-8 kit after the indicated treatments. As shown in Figure 4C, compared with sham-treated cells, IR alone had no effect on cell viability while a significant decrease in cell viability was seen with HT alone treatment. However, IR+HT treatments further reduced cell viability significantly at 48 hours compared to HT alone. Together, the results demonstrate that HT combined with IR is more effective than IR alone in reducing the proliferation and viability of CSCs.

### HT induced radiosensitization was associated with elevated intracellular ROS level in irradiated CSCs

**Figure 5:**
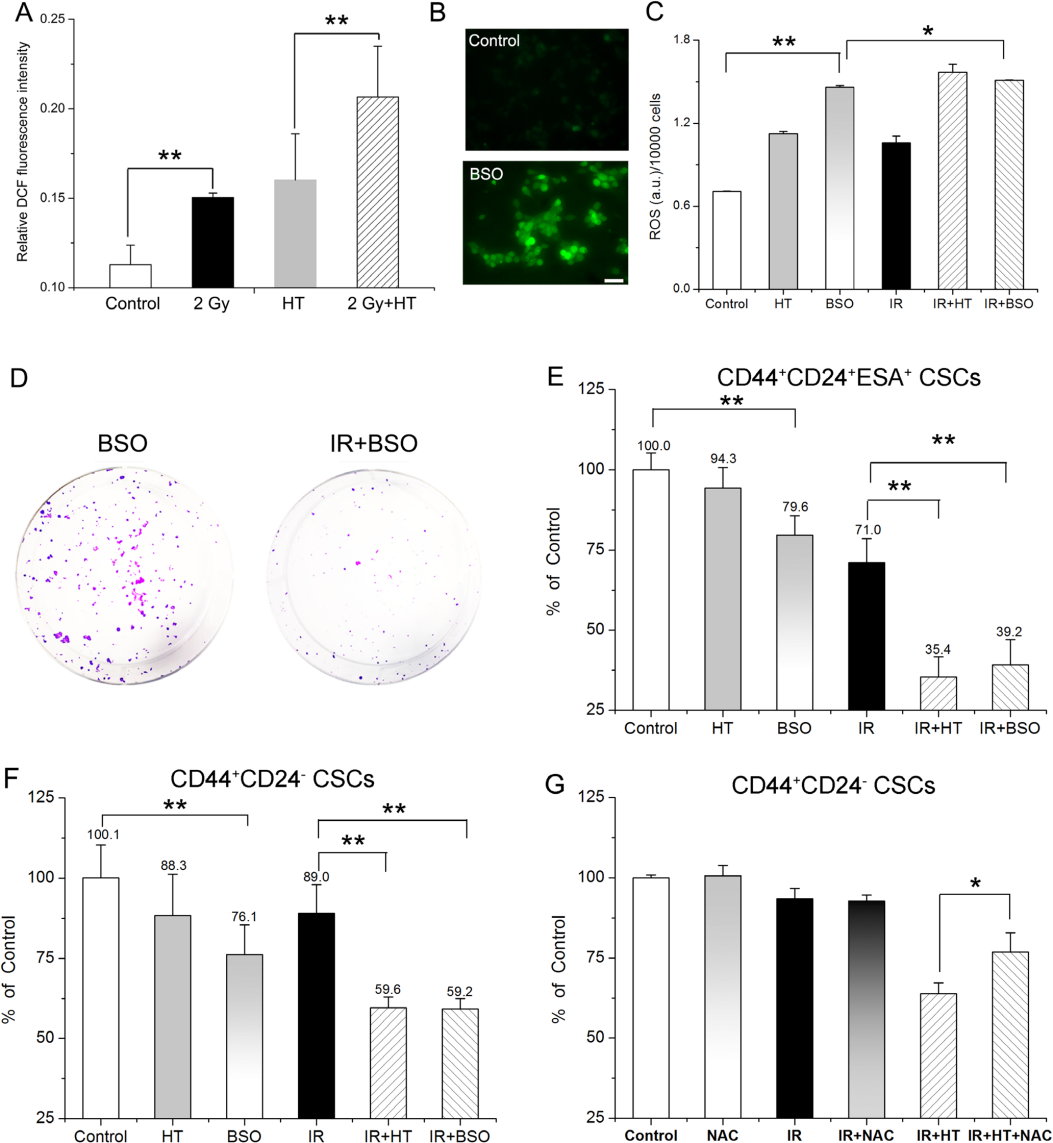
Association of elevated ROS level with HT induced radiosensitization in breast CSCs and pancreatic CSCs (**A**) Intracellular ROS concentrations of CD44^+^CD24^-^ CSCs in control, HT, 2 Gy and 2 Gy+HT treatment groups. No treated group as control. (**B**) Representative images of intracellular ROS concentrations in CD44^+^CD24^–^ CSCs treated with or without 1 mM BSO for 24 hours. Bar = 50 μm. (**C**) Intracellular ROS concentrations of CD44^+^CD24^–^ CSCs in the indicated treatment groups. (**D**) Representative images of colony formation in CD44^+^CD24^+^ESA^+^ CSCs at 14 days after treated with BSO (1 mM) or IR+BSO. (**E**) Colony survival of CD44^+^CD24^+^ESA^+^ CSCs treated with or without 1 mM BSO for 24 hours prior to the indicated treatments. Each data was normalized to that of the sham-treated control. (**F**) CD44^+^CD24^–^ CSCs were cultured as mammospheres with or without 1 mM BSO for 24 hours prior to the indicated treatments. Mammosphere survival of each group was analyzed statistically. Each data was normalized to that of the sham-treated control. (**G**) CD44^+^CD24^–^ CSCs after indicated treatments were cultured as mammospheres in presence or absence with 100 μM NAC. Post 7 days’ culturing, number of mammospheres per 2500 cells were counted and mammosphere survival of each group was analyzed statistically. Each data was normalized to that of the sham-treated control. The results are presented as the mean ± SD, as determined from three independent experiments. ^*^P < 0.05, ^**^P < 0.01.

CSCs in tumors contain lower ROS levels and enhanced ROS scavengers such as glutathione (GSH) compared to their non-tumorigenic progeny, which results in tumor radioresistance [10, 30]. To address whether modulating redox status played a critical role in HT-induced radiosensitization, we detected the intracellular ROS levels in CSCs after the indicated treatments. As shown in Figure 5A, intracellular ROS level increased significantly in all of the three treated groups compared to the control group. Additionally, a further 1.3-fold increase in ROS level was detected in treated with IR combined HT when compared to HT alone or IR alone (Figure 5A). It is suggested that the elevated intracellular ROS level associated with HT induced radiosensitization.

Next, the association was further confirmed by modifying redox status in CSCs bidirectionally. On one hand, buthionine sulphoximine (BSO) increases intracellular ROS level by inhibiting glutamate-cysteine ligase [31–32]. Presently, fluorescence analysis showed that the ROS formation of BSO treated CSCs was significantly higher than that of control (Figure 5B and 5C). Although BSO itself had significant impact on mammosphere and colony formation (Figure 5E and 5F), exposure to 1 mM BSO in CSCs leads to significant radiosensitization, showing significant increase in ROS formation (Figure 5C), remarkable decrease in colony formation (79.6% ± 6.1% for BSO *versus* 39.2% ± 7.9% for IR+BSO, Figure 5D and 5E) and mammosphere formation (76.1% ± 9.3% for BSO *versus* 59.2% ± 3.2% for IR+BSO, Figure 5F). Furthermore, no significant difference in mammosphere formation can be observed between HT alone and BSO treatment (*P* = 0.17, Figure 5F), suggesting BSO mimicked treatment with HT alone. Interestingly, there was no remarkable difference of ROS formation between IR+HT group and IR+BSO group (Figure 5C), suggesting that BSO mimicked HT induced radiosensitivity. Moreover, as shown in Figure 5E, both IR+BSO group and IR+HT group reduced colony formation by additional ~32% compared to IR group (39.2% ± 7.9% for IR+BSO and 35.4% ± 6.3% for IR+HT *versus* 71.0% ± 7.5% for IR). The similar extent (29%) of reducing mammosphere growth was also found in either IR+BSO group or IR+HT group compared with IR group (59.2% ± 3.2% for IR+BSO and 59.6% ± 3.4% for IR+HT *versus* 89.0% ± 9.0% for IR, Figure 5F), Collectively, the results indicated that BSO mimicked HT induced radiosensitivity. Additionally, the effect of HT treatments on antioxidant system in irradiated CSCs was investigated. As shown in Supplementary Figure 3, IR+HT treatment significantly reduced superoxide dismutase (SOD) activity by additional 27% when compared to IR alone, suggesting that the combined treatment resulted in stronger inhibition of the antioxidant activity than with IR alone.

On the other hand, N-acetylcysteine (NAC), an aminothiol and synthetic precursor of intracellular cysteine and GSH, is considered as an important antioxidant [33]. We then tested whether the addition of NAC can neutralize the ROS-induced toxicity for further diminishing the HT induced radiosensitivity in CSCs. As shown in Figure 5G, 100 μM NAC significantly rescued HT induced radiosensitivity, showing increased cell survival (63.9% ± 3.2% in IR+HT group *versus* 76.8% ± 5.9% in IR+HT+NAC group, *P <* 0.05). Moreover, compared to IR+HT group, mammosphere size of IR+HT+NAC group was significantly increased, almost to the level of the control group (84.1 ± 22.9 μm in IR+HT group *versus* 132.2 ± 25.2 μm in IR+HT+NAC group, *P <* 0.01, Supplementary Figure 4). Moreover, significant difference was also found between IR+HT+NAC group and IR+NAC group (Figure 5G, *P <* 0.05). The data demonstrated that NAC can partly but significantly rescued HT induced radiosensitivity. Collectively, these results further demonstrated the association of ROS with HT induced radiosensitivity in CSCs.

## DISCUSSION

Many Phase III clinical trials show the combination of HT with IR result in higher response rates, accompanied with improved local tumor control rates, better palliative effects, and/or better overall survival rates [34–36]. Our study determined that human breast CSCs and pancreatic CSCs can be radiosensitized significantly by HT treatment, showing significant decrease in self-renewal (Figure 3) as well as cell survival and viability both *in vitro* (Figure 4) and *in vivo* (Table 2). Since CSCs are suggested to be the major cause of tumor radio-resistance, dormancy and recurrence after radiotherapy, understanding the mechanism(s) of radiosensitizing CSCs are therefore of fundamental importance. Previous reports suggested that IR+HT may prevent the efficient DNA damage repair in breast CSCs [21], reduce AKT activation and impair proliferation in glioma stem-like cells [22]. In this study, we found HT induced radiosensitization was associated with increased intracellular ROS level in IR+HT treated CSCs (Figure 5). The association was further confirmed bidirectionally under different redox status in CSCs through adding BSO or NAC (Figure 5D–5G).

How does HT treatment result in elevated intracellular ROS level in CSCs? We found that CSCs contained lower ROS level than non-CSCs. This can be achieved by upregulation of free radical scavenger, downregulation of ROS-producing enzymes, reduced mitochondrial mass and low oxygen consumption [10, 37]. Actually, previous studies reported that hyperthermia can not only increase generation of mitochondrial superoxide anion levels [38] and H_2_O_2_ [39], but also inhibit mitochondrial antioxidant system such as SOD (also illustrated in Supplementary Figure 3) [40–41] and GSH [42–43], which may contribute to increase ROS levels in CSCs.

How does HT-induced elevated intracellular ROS level radiosensitize CSCs? On one hand, elevated ROS level may disturb self-renewal of CSCs. As shown in Figure 3, IR+HT treatment significantly decreased colony formation, mammosphere number as well as mammosphere size compared to IR alone, indicating attenuated self-renewal capacity. Atsushi *et al.* found that elevated intracellular ROS level promoted the loss of self-renewal and the differentiation of glioma-initiating cells via ROS-dependent activation of p38 MAPK [44]. McCord *et al.* suggested that hypoxia induces the expression of genes related to stem cell function such as Sox2 and Oct4 [45], indicating the link between intracellular ROS level and cancer stemness.

On the other hand, HT-induced elevated intracellular ROS level may also disturb the process of DNA damage and/or repair in CSCs under IR treatment. Since ROS is a critical mediator in ionizing radiation-induced cell eliminating [46–47], CSCs developed less DNA damage compared to non-CSCs [10, 48]. Additionally, CSCs were suggested to preferentially activate DNA damage checkpoints and repair DNA damage more efficiently than non-CSCs [6]. However, excess ROS could result in DNA-protein cross-linkage and damage to the DNA repair system [49]. Laszlo and Fleischer determined that heat induces perturbations of DNA damage signaling pathways [50]. Together, it is likely that increasing ROS to a threshold that is incompatible with cell viability and targeting the enhanced antioxidant mechanisms could contribute to eliminate CSCs effectively.

In summary, our results suggested that the association of elevated intracellular ROS level and HT induced radiosensitivity in human breast CSCs and pancreatic CSCs. Notably, ROS is a critical mediator in ionizing radiation-induced cell killing [46–47], involving in cell proliferation, survival, motility, angiogenesis and maintenance of tumor stemness [51], a redox-modulating strategy such as HT in combination with conventional radiotherapy may be an attractive approach to improve therapeutic outcomes.

## MATERIALS AND METHODS

### Cell culture

Human breast cancer cell line MCF7 was purchased from American Type Culture Collection. Human pancreatic cancer cell line L3.6pl was kindly provided by Dr Heeschen (Spanish National Cancer Research Centre). Cells were cultured in DMEM high glucose medium with 10% fetal calf serum. Isolated CSCs were cultured in plates precoated with 1% geltrex or as mammospheres with DMEM/F12 containing N2, B27, 10 ng/ml bFGF and 20 ng/ml EGF as described previously [23, 48, 52].

### Prospective isolation of putative CSCs

Cells were trypsinized into single cell suspension and were counted. For staining, samples were usually incubated with antibodies for 30 minutes at 4°C. Unbound antibody was washed off and cells were sorted by flow cytometry no longer than 30 min post staining on a BD Aria III. In order to isolate breast CSCs, the antibodies used were anti-CD44 PE and anti-CD24 FITC (BD Pharmingen). The purity of isolated breast CSCs was determined by standard flow cytometry analysis. The purity of isolated CD44^+^CD24^–^ CSCs regularly exceeded 98%. As for sorting pancreatic CSCs, cells were stained with APC-labeled CD44 antibodies and PE-labeled CD24 antibodies (BD Pharmingen), as well as BV421-labeled ESA antibodies (Biolegend). The triple positive (CD44^+^CD24^+^ESA^+^) cells and L3.6pl were reanalyzed by flow cytometry analysis using PE-labeled Sox2 antibodies (Biolegend). In sorted cell implantation experiments, CSCs were isolated using APC-labeled antibody against human CD133 (Miltenyi Biotech).

### Cell treatments

For HT treatment, cells were incubated in a 43°C humidified incubator, 5% CO_2_. For IR+HT treatments, the same HT treatment procedure was performed immediately after irradiation. The optimal duration of heating was 1.5 hours for pancreatic CSCs and 2 hours for breast CSCs. For irradiation, cells were irradiated by γ-rays from a 2.6 × 10^5^ Curie ^60^Co source at Peking University with total dose of 0, 1, 2, 3, 4, 6, 8, 9 and 12 Gy (dose rate 0.94 Gy/min). BSO and NAC were purchased from Sigma. For BSO treatment, there were two groups of cells. One group was pretreated with 1mM BSO for 24 hours and the other one was as the BSO negative group. Then, both two groups were treated with or without IR, HT or IR+HT. After treatment, all groups were cultured as described in Mammosphere formation assay or in Clonogenic assay. For NAC treatment, cells were non-treated or treated with IR, HT or IR+HT. Then, half of each group was cultured as mammospheres in medium contained with 100 μM NAC for 7 days. The remaining half was as the corresponding negative comparison of each group.

### Mammosphere formation assay

Mammosphere formation assay was performed as described previously [5]. In brief, either 2500 cells/well or 1000 cells/well was grown in 24-well low-attachment plates. After 7 days, all mammospheres in each well were assessed via bright-field microscopy (Zeiss). Mammospheres were imaged and analyzed with ImageJ software to obtain the diameters. The mammospheres which diameter was larger than 50 μm were counted. Three independent experiments were performed.

### ROS assay

Intracellular ROS was assayed as described previously [10]. In brief, cells at confluency were incubated with 2 μM CM-H2DCFDA (Invitrogen) and treated with HT, IR, or IR+HT. After treatments in the indicated groups, ROS concentration was detected immediately using a fluorescence reader (Varioskan flash, Thermo scientific). In other experiments, cells were pretreated with 1 mM BSO for 24 hours, and then incubated with 2 μM CM-H2DCFDA. The fluorescence was measured in a fluorescence plate reader and was imaged by fluorescence microscopy (Zeiss). Three independent experiments were performed.

### Cell viability

Cell viability was assayed using cell counting kit-8 (CCK-8, DOJINDO) according to the manufacturer's instructions. For cell number analysis, cells were fixed (4% paraformaldehyde) and stained with Hochest33342 (Dojindo Molecular Technologies, Inc) at 24, 48 and 72 hours after treatments, and then imaged as described previously [21]. Three independent experiments were performed.

### Colony forming assay

Clonogenic assays were performed immediately after treatments by plating cells into triplicate 6-well cell culture plates. After 14 days, cells were fixed with 4% paraformaldehyde and stained with 1% crystal violet, and colonies containing more than 50 cells were counted. Three independent experiments were performed.

### Sorted cell implantation into BALB/c nude mice

Sorted cells (CD133^+^ *versus* CD133^-^) were washed with serum-free HBSS after flow cytometry and suspended in serum free-DF12/Matrigel mixture (1:1 volume) followed by injection s.c. into the right and left armpit using a 26-gauge needle (n ≥ 5 animals per group). Cell number of each injection was 500, 1000 or 5000. Animals underwent autopsy at 40 days after cell implantation. In other experiments, 5000 CD133^+^ cells were non-treated or treated with IR, HT (43°C for 1.5 hours) or IR+HT. Then the former two groups were injected s.c.into the right and left armpit of the same mice. The rest two groups were injected s.c.into another mice (*n* = 6 animals per group). All animal studies and protocols were approved by the Peking University Animal Care committee according to Peking University animal use guidelines.

### Determination of intracellular SOD activity

After the indicated treatments, total SOD activities of the samples was determined using the Total Superoxide Dismutase Assay Kit with WST-8 (Beyotime Institute of Biotechnology, Jiangsu, China) based on the protocols provided by the manufacturer.

### Statistical analysis

The results were presented as mean ± SD. Significance was assessed using Student's *t*-test and chi-square analysis, where appropriate, and defined as *P* < 0.05 or *P* < 0.01 (extremely significant difference).

## SUPPLEMENTARY MATERIALS FIGURES



## References

[1] Schepers AG, Snippert HJ, Stange DE, van den Born M, van Es JH, van de Wetering M, Clevers H (2012). Lineage Tracing Reveals Lgr5+ Stem Cell Activity in Mouse Intestinal Adenomas. Science.

[2] Driessens G, Beck B, Caauwe A, Simons BD, Blanpain C (2012). Defining the mode of tumour growth by clonal analysis. Nature.

[3] Chen J, Li Y, Yu TS, McKay RM, Burns DK, Kernie SG, Parada LF (2012). A restricted cell population propagates glioblastoma growth after chemotherapy. Nature.

[4] Woodward WA, Chen MS, Behbod F, Alfaro MP, Buchholz TA, Rosen JM (2007). WNT/β-catenin mediates radiation resistance of mouse mammary progenitor cells. Proc Natl Acad Sci USA.

[5] Phillips TM, McBride WH, Pajonk F (2006). The response of CD24(-/low)/CD44+ breast cancer-initiating cells to radiation. J Natl Cancer Inst.

[6] Bao S, Wu Q, McLendon RE, Hao Y, Shi Q, Hjelmeland AB, Dewhirst MW, Bigner DD, Rich JN (2006). Glioma stem cells promote radioresistance by preferential activation of the DNA damage response. Nature.

[7] Schatton T, Murphy GF, Frank NY, Yamaura K, Waaga-Gasser AM, Gasser M, Zhan Q, Jordan S, Duncan LM, Weishaupt C, Fuhlbrigge RC, Kupper TS, Sayegh MH (2008). Identification of cells initiating human melanomas. Nature.

[8] Fillmore CM, Kuperwasser C (2008). Human breast cancer cell lines contain stem-like cells that self-renew, give rise to phenotypically diverse progeny and survive chemotherapy. Breast Cancer Res.

[9] Eramo A, Ricci-Vitiani L, Zeuner A, Pallini R, Lotti F, Sette G, Pilozzi E, Larocca LM, Peschle C, De Maria R (2006). Chemotherapy resistance of glioblastoma stem cells. Cell Death Differ.

[10] Diehn M, Cho RW, Lobo NA, Kalisky T, Dorie MJ, Kulp AN, Qian D, Lam JS, Ailles LE, Wong M, Joshua B, Kaplan MJ, Wapnir I (2009). Association of reactive oxygen species levels and radioresistance in cancer stem cells. Nature.

[11] Vlashi E, Kim K, Lagadec C, Donna LD, McDonald JT, Eghbali M, Sayre JW, Stefani E, McBride W, Pajonk F (2009). *In Vivo* Imaging, Tracking, and Targeting of Cancer Stem Cells. J Natl Cancer Inst.

[12] van der Zee J, González D, van Rhoon GC, van Dijk JD, van Putten WL, Hart AA (2000). Comparison of radiotherapy alone with radiotherapy plus hyperthermia in locally advanced pelvic tumours: a prospective, randomised, multicentre trial. The Lancet.

[13] Vernon CC, Hand JW, Field SB, Machin D, Whaley JB, van der Zee J, van Putten WL, van Rhoon GC, van Dijk JD, González González D, Liu FF, Goodman P, Sherar M (1996). Radiotherapy with or without hyperthermia in the treatment of superficial localized breast cancer: Results from five randomized controlled trials. Int J Radiat Oncol Biol Phys.

[14] Emami B, Scott C, Perez CA, Asbell S, Swift P, Grigsby P, Montesano A, Rubin P, Curran W, Delrowe J, Arastu H, Fu K, Moros E (1996). Phase III study of interstitial thermoradiotherapy compared with interstitial radiotherapy alone in the treatment of recurrent or persistent human tumors: A prospectively controlled randomized study by the radiation therapy oncology group. Int J Radiat Oncol Biol Phys.

[15] van der Zee J, Vujaskovic Z, Kondo M, Sugahara T (2008). The Kadota Fund International Forum 2004–Clinical group consensus. Int J Hyperthermia.

[16] Horsman MR, Overgaard J (2007). Hyperthermia: a Potent Enhancer of Radiotherapy. Clin Oncol.

[17] Wust P, Hildebrandt B, Sreenivasa G, Rau B, Gellermann J, Riess H, Felix R, Schlag PM (2002). Hyperthermia in combined treatment of cancer. The Lancet Oncology.

[18] Crezee H, van Leeuwen CM, Oei AL, Stalpers LJ, Bel A, Franken NA, Kok HP (2016). Thermoradiotherapy planning: Integration in routine clinical practice. Int J Hyperthermia.

[19] Sapareto SA, Peter Raaphorst G, Dewey WC (1979). Cell killing and the sequencing of hyperthermia and radiation. Int J Radiat Oncol Biol Phys.

[20] Dewhirst MW, Vujaskovic Z, Jones E, Thrall D (2005). Re-setting the biologic rationale for thermal therapy. Int J Hyperthermia.

[21] Atkinson RL, Zhang M, Diagaradjane P, Peddibhotla S, Contreras A, Hilsenbeck SG, Woodward WA, Krishnan S, Chang JC, Rosen JM (2010). Thermal Enhancement with Optically Activated Gold. Nanoshells Sensitizes Breast Cancer Stem Cells to Radiation Therapy. Sci Transl Med.

[22] Man J, Shoemake JD, Ma T, Rizzo AE, Godley AR, Wu Q, Mohammadi AM, Bao S, Rich JN, Yu JS (2015). Hyperthermia Sensitizes Glioma Stem-like Cells to Radiation By Inhibiting AKT Signaling. Cancer Res.

[23] Yang G, Quan Y, Wang W, Fu Q, Wu J, Mei T, Li J, Tang Y, Luo C, Ouyang Q, Chen S, Wu L, Hei TK (2012). Dynamic equilibrium between cancer stem cells and non-stem cancer cells in human SW620 and MCF-7 cancer cell populations. Br J Cancer.

[24] Neve RM, Chin K, Fridlyand J, Yeh J, Baehner FL, Fevr T, Clark L, Bayani N, Coppe JP, Tong F, Speed T, Spellman PT, DeVries S (2006). A collection of breast cancer cell lines for the study of functionally distinct cancer subtypes. Cancer Cell.

[25] Shipitsin M, Campbell LL, Argani P, Weremowicz S, Bloushtain-Qimron N, Yao J, Nikolskaya T, Serebryiskaya T, Beroukhim R, Hu M, Halushka MK, Sukumar S, Parker LM (2007). Molecular Definition of Breast Tumor Heterogeneity. Cancer Cell.

[26] Heidt DG, Li C, Mollenberg N, Clarke MF, Simeone D (2006). Identification of pancreatic cancer stem cells. J Surg Res.

[27] Hermann PC, Huber SL, Herrler T, Aicher A, Ellwart JW, Guba M, Bruns CJ, Heeschen C (2007). Distinct Populations of Cancer Stem Cells Determine Tumor Growth and Metastatic Activity in Human Pancreatic Cancer. Cell Stem Cell.

[28] Albright N (1987). Computer Programs for the Analysis of Cellular Survival Data. Radiat Res.

[29] Herreros-Villanueva M, Zhang JS, Koenig A, Abel EV, Smyrk TC, Bamlet WR, de Narvajas AA, Gomez TS, Simeone DM, Bujanda L, Billadeau DD (2013). SOX2 promotes dedifferentiation and imparts stem cell-like features to pancreatic cancer cells. Oncogenesis.

[30] Nomura A, Dauer P, Gupta V, McGinn O, Arora N, Majumdar K, Uhlrich C, Dalluge J, Dudeja V, Saluja A, Banerjee S (2016). Microenvironment mediated alterations to metabolic pathways confer increased chemo-resistance in CD133+ tumor initiating cells. Oncotarget.

[31] Bailey HH (1998). l-S,R-buthionine sulfoximine: historical development and clinical issues. Chem Biol Interact.

[32] Burhans WC, Heintz NH (2009). The cell cycle is a redox cycle: Linking phase-specific targets to cell fate. Free Radical Biol Med.

[33] Zafarullah M, Li WQ, Sylvester J, Ahmad M (2003). Molecular mechanisms of N-acetylcysteine actions. Cell Mol Life Sci.

[34] Jones EL, Oleson JR, Prosnitz LR, Samulski TV, Vujaskovic Z, Yu D, Sanders LL, Dewhirst MW (2005). Randomized Trial of Hyperthermia and Radiation for Superficial Tumors. J Clin Oncol.

[35] Franckena M, Lutgens LC, Koper PC, Kleynen CE, van der Steen-Banasik EM, Jobsen JJ, Leer JW, Creutzberg CL, Dielwart MF, van Norden Y, Canters RA, van Rhoon GC, van der Zee J (2009). Radiotherapy and Hyperthermia for Treatment of Primary Locally Advanced Cervix Cancer: Results in 378 Patients. Int J Radiat Oncol Biol Phys.

[36] Gabriele P, Ferrara T, Baiotto B, Garibaldi E, Marini PG, Penduzzu G, Giovannini V, Bardati F, Guiot C (2009). Radio hyperthermia for re-treatment of superficial tumours. Int J Hyperthermia.

[37] Ye XQ, Li Q, Wang GH, Sun FF, Huang GJ, Bian XW, Yu SC, Qian GS (2011). Mitochondrial and energy metabolism-related properties as novel indicators of lung cancer stem cells. Int J Cancer.

[38] Mujahid A, Sato K, Akiba Y, Toyomizu M (2006). Acute heat stress stimulates mitochondrial superoxide production in broiler skeletal muscle, possibly via downregulation of uncoupling protein content. Poult Sci.

[39] Powers RH, Stadnicka A, Kalbfleish JH, Skibba JL (1992). Involvement of Xanthine Oxidase in Oxidative Stress and Iron Release during Hyperthermic Rat Liver Perfusion. Cancer Res.

[40] Moriyama-Gonda N, Igawa M, Shiina H, Urakami S, Shigeno K, Terashima M (2002). Modulation of heat-induced cell death in PC-3 prostate cancer cells by the antioxidant inhibitor diethyldithiocarbamate. BJU Int.

[41] Morrison JP, Coleman MC, Aunan ES, Walsh SA, Spitz DR, Kregel KC (2005). Aging reduces responsiveness to BSO- and heat stress-induced perturbations of glutathione and antioxidant enzymes. Am J Physiol Regul Integr Comp Physiol.

[42] Mitchell JB, Russo A, Kinsella TJ, Glatstein E (1983). Glutathione Elevation during Thermotolerance Induction and Thermosensitization by Glutathione Depletion. Cancer Res.

[43] Zhao QL, Fujiwara Y, Kondo T (2006). Mechanism of cell death induction by nitroxide and hyperthermia. Free Radical Biol Med.

[44] Sato A, Okada M, Shibuya K, Watanabe E, Seino S, Narita Y, Shibui S, Kayama T, Kitanaka C (2014). Pivotal role for ROS activation of p38 MAPK in the control of differentiation and tumor-initiating capacity of glioma-initiating cells. Stem Cell Res.

[45] McCord AM, Jamal M, Shankavarum UT, Lang FF, Camphausen K, Tofilon PJ (2009). Physiologic Oxygen Concentration Enhances the Stem-Like Properties of CD133+ Human Glioblastoma Cells *In vitro*. Mol Cancer Res.

[46] Ward JF (1985). Biochemistry of DNA Lesions. Radiat Res Suppl.

[47] Powell S, McMillan TJ (1990). DNA damage and repair following treatment with ionizing radiation. Radiother Oncol.

[48] Fu Q, Quan Y, Wang W, Mei T, Wu J, Li J, Yang G, Ren X, Xue J, Wang Y (2012). Response of cancer stem-like cells and non-stem cancer cells to proton and γ-ray irradiation. Nucl Instrum Meth B.

[49] Packeisen J, Kaup-Franzen C, Knieriem HJ (1999). Detection of surface antigen 17–1A in breast and colorectal cancer. Hybridoma.

[50] Laszlo A, Fleischer I (2009). Heat-Induced Perturbations of DNA Damage Signaling Pathways are Modulated by Molecular Chaperones. Cancer Res.

[51] Kobayashi CI, Suda T (2012). Regulation of reactive oxygen species in stem cells and cancer stem cells. J Cell Physiol.

[52] Pollard SM, Yoshikawa K, Clarke ID, Danovi D, Stricker S, Russell R, Bayani J, Head R, Lee M, Bernstein M, Squire JA, Smith A, Dirks P (2009). Glioma Stem Cell Lines Expanded in Adherent Culture Have Tumor-Specific Phenotypes and Are Suitable for Chemical and Genetic Screens. Cell Stem Cell.

