# Supplementary material for: Association of elevated reactive oxygen species and hyperthermia induced radiosensitivity in cancer stem-like cells
Source: Oncotarget. 2017 Oct 9;8(60):101560–71. doi: 10.18632/oncotarget.21678 (PMC5731896; doi:10.18632/oncotarget.21678)
Supplement: Supplementary file 1 [file oncotarget-08-101560-s001.pdf]

Association of elevated reactive oxygen species and hyperthermia induced radiosensitivity in cancer stem-like cells

SUPPLEMENTARY MATERIALS

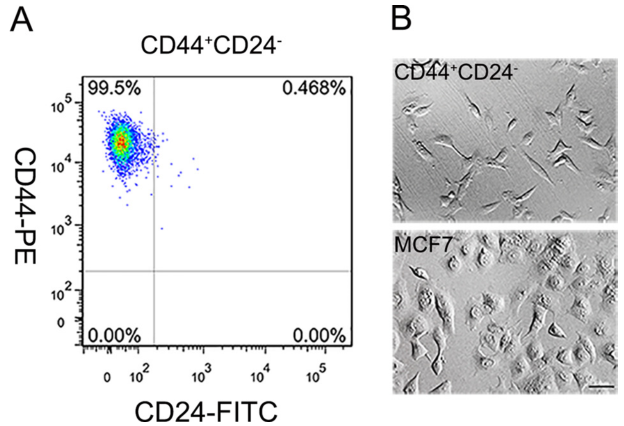

Supplementary Figure 1: Reanalysis of sorted CD44$^+$CD24$^-$ CSCs. (**A**) After 1 day cultivation of CD44$^+$CD24$^-$ CSCs, the purity of these cells were reanalyzed by flow cytometry. (**B**) Cell morphology of CD44$^+$CD24$^-$ CSCs and MCF7 cells imaged by 10x objective (Bar = 100 µm).

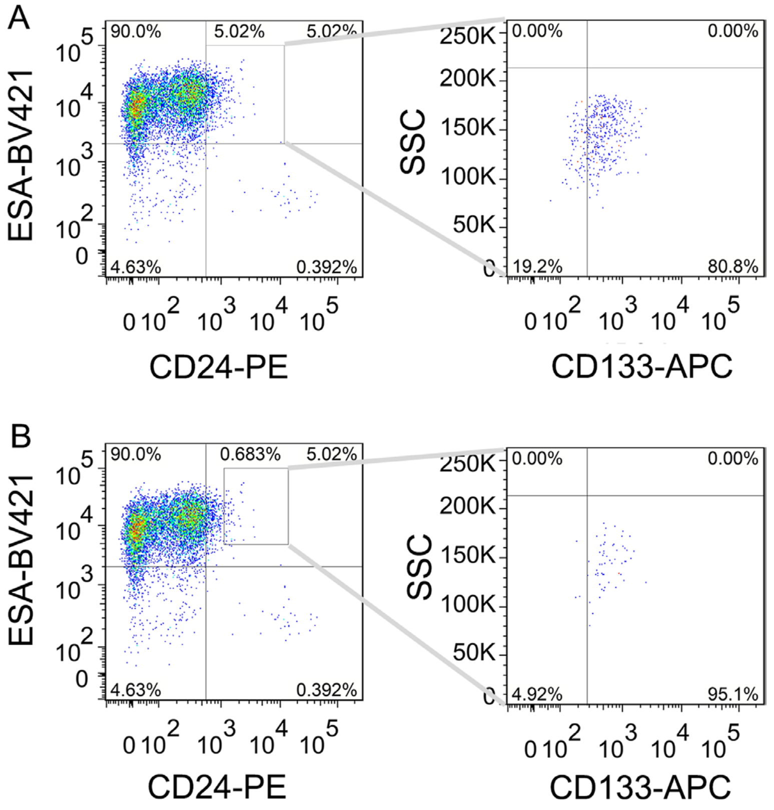

Supplementary Figure 2: Flow cytometry analysis of the pattern of CD133 staining in CD44$^+$CD24$^+$ESA$^+$ CSCs. Since all L3.6pl cells were positive for CD44 (Figure 1B), the analysis of the CD133 content was restricted to CD24$^+$ESA$^+$ cells. (**A**) Flow cytometry analysis to measure ESA and CD24 expression of L3.6pl cells (Left) and the patterns of CD133 staining of CD24$^+$ESA$^+$ cells as shown in the frame (Right). (**B**) The pattern of CD133 staining of the brighter CD24$^+$ESA$^+$ cells as shown in the frame.

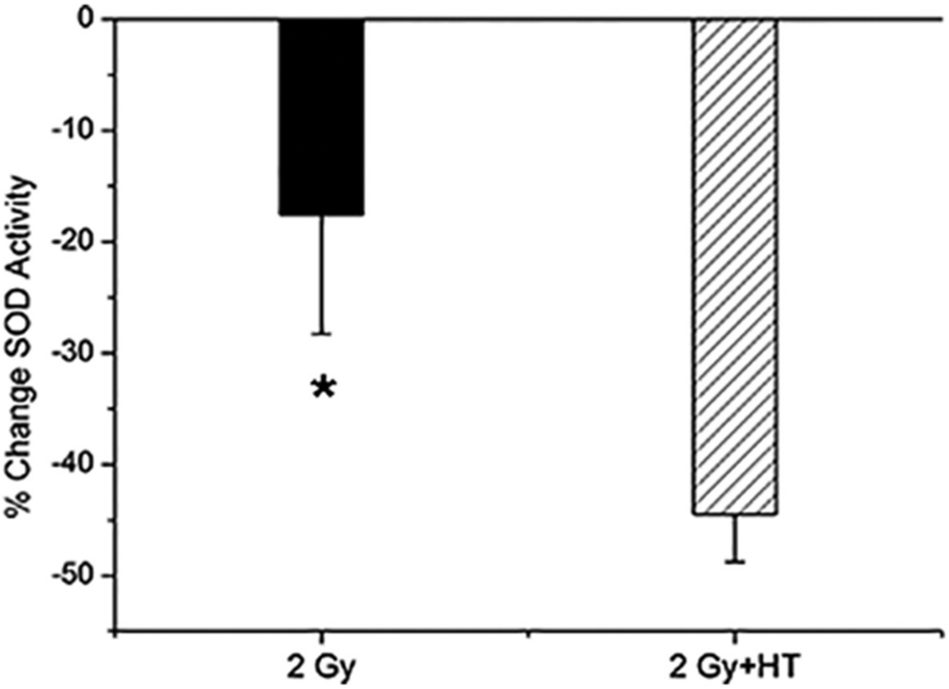

Supplementary Figure 3: Effect of the indicated treatments on SOD activity in CD44⁺CD24⁻ CSCs. Effect of IR (2 Gy) alone and IR+HT (2 Gy irradiation followed by 43°C for 2 hours) on SOD activity. SOD activity was determined by WST-8 assay. Each data was normalized to that of the sham-treated control. The results are presented as the mean ± SD, as determined from three independent experiments. $^{*}P < 0.05$.

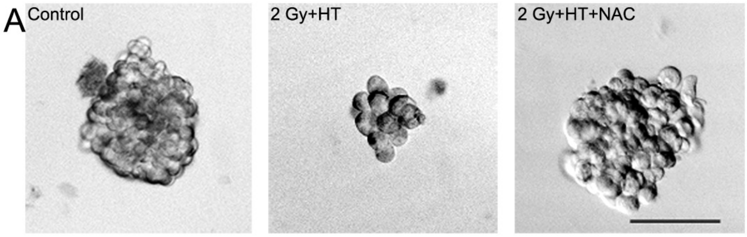

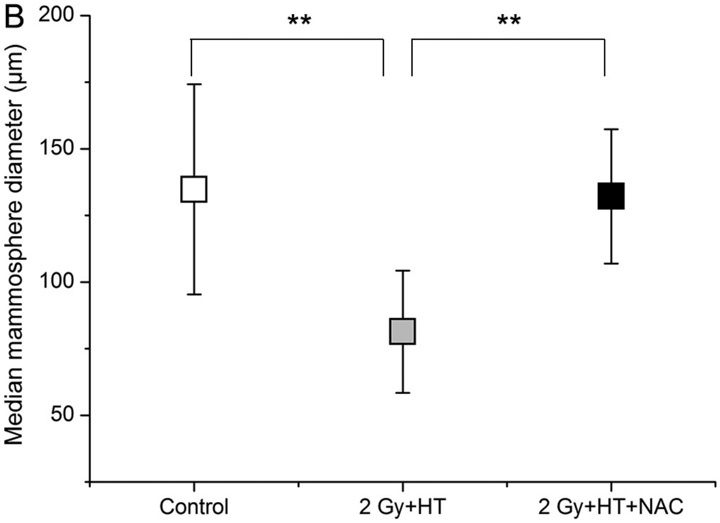

Supplementary Figure 4: Characterization of mammospheres derived from CD44⁺CD24⁻ CSCs after the indicated treatments. (**A–B**) Representative images of mammosphere formation in CD44⁺CD24⁻ CSCs at day 7 after treated as described in Figure 5G, Bar = 100 μm (A) and mean values for mammosphere diameters of the indicated treatment groups (B). At least 40 mammospheres were measured in each group. The results are presented as the mean ± SD, as determined from three independent experiments. $^{**}P < 0.01$.
